# Iterative spectral methods for Hamilton-Jacobi-Bellman quasi-variational inequality in finance

**DOI:** 10.1371/journal.pone.0359303

**Published:** 2026-09-30

**Authors:** Minlan Lei, Zhengyang Lu

**Affiliations:** School of Mathematics, Southwestern University of Finance and Economics, Chengdu, Sichuan, China; Lahore School of Economics, PAKISTAN

## Abstract

This study proposes a novel computational scheme for utility-maximization problems involving optimal stopping, formulated as Hamilton-Jacobi-Bellman quasi-variational inequalities. The methodology integrates Gauss-Lobatto-Legendre spectral discretization with a penalization method and is solved efficiently via policy iteration. We establish the convergence of the penalized scheme and verify the effectiveness and robustness of the framework through a series of numerical experiments.

## Introduction

In the broader context of financial optimization, Hamilton-Jacobi-Bellman (HJB) equations have become the fundamental tool for addressing complex dynamic decision-making problems, ranging from classic continuous-time portfolio selection [1] to general stochastic control theories [2]. This paper centers on utility maximization problems embedded with optimal stopping constraints, for which a series of theoretical studies have been developed over the past decades. Early foundational research was carried out by [3] and [4]. Later [5], and [6] analyzed the existence and equivalence of solutions, yet their work failed to provide a complete characterization of the free boundary. To remedy this limitation [7], and [8] adopted dual transformation techniques to convert the original problems into standard free boundary problems, while [9] proposed a global approximation strategy.

Beyond these classical theoretical settings, the application of continuous-time differential models and boundary value problems has grown increasingly sophisticated, driving recent breakthroughs across diverse scientific domains. Such advanced continuous models have not only provided profound new insights into complex biological systems, such as population dynamics [10], but are also being actively deployed to solve intricate real-world financial problems, including optimal investment considerations for life insurance portfolios [11]. To construct reliable optimization models that meet these practical demands, incorporating realistic market dynamics—such as stochastic volatility, leverage effects, and long-memory features—is essential. Traditional constant-volatility frameworks represented by the classical Black-Scholes model enjoy analytical tractability but fail to reproduce typical empirical patterns like volatility smiles and return skewness. To overcome this critical defect, stochastic volatility models were first proposed by [12] and further extended to high-precision numerical and regime-switching frameworks in [13], evolving into the mainstream industry standard. Building on this foundation, the literature on non-constant volatility has recently expanded significantly to capture even more complex empirical features. For instance, extensive empirical evidence has shown that volatility time series exhibit rough behavior, leading to the rapid development of rough volatility models [14]. Furthermore, optimal portfolio selection in complex stochastic environments, such as those with fast mean-reverting or fractional volatility dynamics, continues to attract substantial academic interest [15].

Unfortunately, incorporating stochastic volatility features transforms the original HJB equations into degenerate multidimensional quasi-variational inequalities (QVIs), which pose severe theoretical and computational difficulties. Computationally, solving optimal stopping and stochastic control problems in these advanced stochastic volatility frameworks has seen a recent surge in machine learning techniques, such as neural-network-based HJB solvers [16] and deep optimal stopping algorithms [17]. While these deep learning approaches scale exceptionally well to high-dimensional problems, they often lack rigorous convergence analysis and struggle to provide sharp, highly accurate resolutions of the early exercise boundary. On the other hand, for low-to-medium dimensional problems such as the 2D Heston-type QVI, resolving the free boundary with mathematical rigor remains crucial. Conventional numerical discretization approaches, such as finite difference and finite element methods (see [18–20]), are frequently hampered by the curse of dimensionality and severe grid oscillations near free boundaries. Recent research has constructed refined penalty formulations and structure-preserving algorithms to cope with complicated financial constraints and regime shifts [13, 21], as well as the spectral control frameworks established in [22]. Despite these methodological advances, it remains an unresolved and challenging task to achieve high-order convergence rates and accurate free-boundary resolution for multi-dimensional Heston-type utility maximization problems subject to optimal stopping constraints. Given the inherent complexity of the Heston framework, we first develop and validate our numerical scheme in the simpler Black-Scholes setting, and then extend it to the Heston model.

To address this research gap and bridge the trade-off between high accuracy and computational efficiency, the present paper develops a discretization scheme based on Gauss-Lobatto-Legendre (GLL) spectral elements. By taking full advantage of the inherent high-order accuracy and outstanding approximation performance of spectral methods, the proposed scheme achieves faster convergence and more precise free boundary identification compared with traditional finite difference discretizations.

The remainder of this paper is organized as follows. Section 2 presents the theoretical framework and numerical formulation of the proposed method. We first formulate the HJB variational inequality for the utility maximization problem under the Black-Scholes model. On this basis, the penalty method is adopted to reformulate the problem and the corresponding convergence results are established. This section also introduces the GLL spectral discretization scheme for the penalized HJB equation and the implementation of the policy iteration algorithm. Additionally, an extended framework based on the Heston model is formulated, and the corresponding discrete scheme is constructed. Section 3 provides numerical experiments to demonstrate the efficiency and improved convergence performance of the proposed scheme. Section 4 concludes the paper with a summary of the main results and research implications.

## Problem formulation and numerical scheme

### Model formulation under the Black-Scholes framework

In this paper we consider a fixed time horizon [0,*T*] with 0<T<+∞. Let (Ω,ℱ,P) be a complete probability space, *W* be a standard Brownian motion, and let $\{{ℱ}_{t}{\}}_{t\in [0,T]}$ be the filtration generated by *W* completed with all *P*-null sets. Assume the financial market consists of one risk-free bond with dynamic price *B* and one risky stock with dynamic price *S*. The bond price process *B* is assumed to follow the process


$$\begin{array}{c} dB_{\tau }=rB_{\tau }d\tau ,\;0\leq \tau \leq T, \end{array}$$

where *r* > 0 is risk-free interest rate. The stock price process *S* follows


$$\begin{array}{c} dS_{\tau }=S_{\tau }(\mu d\tau +\sigma dW_{\tau }),\;0\leq \tau \leq T, \end{array}$$ (1)

where μ>0 and σ>0 are return and volatility rates of the risky asset. The wealth process is given by


$$\begin{array}{c} \frac{dX_{\tau }}{X_{\tau }}=\left[\pi_{\tau }\frac{dS_{\tau }}{S_{\tau }}+(1-\pi_{\tau })\frac{dB_{\tau }}{B_{\tau }}\right], \end{array}$$

where $\pi_{\tau }$ is a progressively measurable control process and represents the proportion of wealth $X_{\tau }$ invested in risky asset $S_{\tau }$. The wealth process is


$$\begin{array}{c} dX_{\tau }=X_{\tau }[rd\tau +\pi_{\tau }\sigma (\theta d\tau +dW_{\tau })],\;0\leq \tau \leq T, \end{array}$$

where $\theta :=\frac{\mu -r}{\sigma }$ denotes the market price of risk. Let 𝒜 denote the set of all admissible progressively measurable investment strategies, and let 𝒯 denote the set of all stopping times adapted to $\{{ℱ}_{t}{\}}_{t\in [0,T]}$. A random time ς is a valid stopping time if $\{\varsigma \leq t\}\in {ℱ}_{t}$ for all t∈[0,T]. The value function is defined as the following constrained expected utility maximization problem:


$$\begin{array}{c} \tilde{V}(\tau ,x):=\underset{\pi \in 𝒜,\;\varsigma \in 𝒯}{\sup}{𝔼}_{\tau ,x}\left[e^{-\beta (\varsigma -\tau )}U(X_{\tau ,x}^{\varsigma }-K)\right], \end{array}$$ (2)

where ${𝔼}_{\tau ,x}[\cdot ]:=𝔼[\cdot |X_{\tau }=x]$, *U* is a utility function that is continuous, strictly increasing and strictly concave on [0,∞), β>0 is the utility discount factor, and K≥0 is the minimum wealth threshold. By the dynamic programming principle, it can be deduced that $\tilde{V}$ satisfies the HJB variational inequality,


$$\begin{array}{c} \min\left\{-{\tilde{V}}_{\tau }(\tau ,x)-\underset{\pi }{\sup}{ℒ}^{\pi }\tilde{V},\tilde{V}-U(x-K)\right\}=0, \end{array}$$ (3)

∀(τ,x)∈(0,T)×(0,∞), where


$$\begin{array}{c} {ℒ}^{\pi }\tilde{V}=\frac{1}{2}\sigma^{2}\pi^{2}x^{2}\cdot {\tilde{V}}_{xx}(\tau ,x)+[\pi (\mu -r)+r]x\cdot {\tilde{V}}_{x}(\tau ,x)-\beta \tilde{V}. \end{array}$$

By the dynamic programming principle, it can be deduced that $\tilde{V}$ satisfies the HJB variational inequality,


$$\begin{array}{c} \min\left\{-{\tilde{V}}_{\tau }(\tau ,x)-\underset{\pi }{\sup}{ℒ}^{\pi }\tilde{V},\tilde{V}-U(x-K)\right\}=0, \end{array}$$ (4)

∀(τ,x)∈(0,T)×(0,∞), where


$$\begin{array}{c} {ℒ}^{\pi }\tilde{V}=\frac{1}{2}\sigma^{2}\pi^{2}x^{2}\cdot {\tilde{V}}_{xx}(\tau ,x)+[\pi (\mu -r)+r]x\cdot {\tilde{V}}_{x}(\tau ,x)-\beta \tilde{V}. \end{array}$$

Using variable transformation t=T−τ, $\tilde{V}(\tau ,x)=\tilde{V}(T-t,x)\equiv \hat{V}(t,x)$, and $\hat{V}(t,x)$ satisfies the following system of HJB variational inequality:


$$\begin{array}{c} \min\left\{{\hat{V}}_{t}(t,x)-\underset{\pi }{\sup}{ℒ}^{\pi }\hat{V},\hat{V}-U(x-K)\right\}=0, \end{array}$$ (5)

∀(t,x)∈(0,T)×(0,∞), where


$$\begin{array}{c} {ℒ}^{\pi }\hat{V}=\frac{1}{2}\sigma^{2}\pi^{2}x^{2}\cdot {\hat{V}}_{xx}(t,x)+[\pi (\mu -r)+r]x\cdot {\hat{V}}_{x}(t,x)-\beta \hat{V}, \end{array}$$ (6)

with the terminal and boundary conditions


$$\begin{array}{cc} & \hat{V}(0,x)=U(x-K),\;x\in \Omega :=(K,x_{\max}),\\ & \hat{V}(t,K)=0,\;t\in [0,T],\\ & \hat{V}(t,x_{\max})=\Psi (t,x_{\max}),\;t\in [0,T]. \end{array}$$ (7)

### Penalty reformulation and convergence analysis

In this section, we transform the HJB variational inequality (5) into a penalized HJB equation using the penalty method (see, e.g.[23–25]). We further demonstrate the convergence of the viscosity solution of the resulting penalized equation to that of the original HJB variational inequality.

We define the penalized HJB equation for the HJB variational inequalitie (5) as follows:


$$\begin{array}{c} V_{t}(t,x)-\underset{\pi }{\sup}{ℒ}^{\pi }V-\lambda (U(x-K)-V(t,x){)}^{+}=0, \end{array}$$ (8)

on [0,T]×Ω, and the terminal and boundary conditions are given by


$$\begin{array}{cc} & V(0,x)=U(x-K),\;x\in \Omega \\ & V(t,K)=0,\;t\in [0,T],\\ & V(t,x_{\max})=\Psi (t,x_{\max})\;t\in [0,T], \end{array}$$ (9)

where $\left(a{)}^{+}:=\max(a,0\right)$ and λ>0.

For ease of analysis, we write (43) as


$$\begin{array}{c} \partial_{t}V(t,x)-ℋV(t,x)=0.\;(t,x)\in (0,T)\times \Omega , \end{array}$$ (10)

where


$$\begin{array}{c} ℋV(t,x):=\underset{\pi }{\sup}{ℒ}^{\pi }V+\lambda (U(x-K)-V(t,x){)}^{+}. \end{array}$$ (11)

Subsequently, we establish the convergence of the viscosity solution to the penalized HJB equation (43) to that of the original variational inequality (5) as the penalty parameter approaches zero.

For defining the viscosity solution to the HJB variational equations (5), it is useful to express it in the form:


$$\begin{array}{c} ℱ({\hat{V}}_{xx}(t,x),{\hat{V}}_{x}(t,x),{\hat{V}}_{t}(t,x),\hat{V}(t,x),t,x)=0. \end{array}$$ (12)

Since the HJB variational inequality (5) is fully nonlinear and degenerate, a classical smooth solution may fail to exist. We therefore work within the framework of viscosity solutions, which is the standard notion of weak solutions for such equations; see [2, Chapter 4] for a comprehensive treatment in the context of stochastic control. The penalized HJB equation (10), being a uniformly elliptic second-order nonlinear PDE, admits a unique viscosity solution, whose existence and uniqueness are guaranteed by the comparison principle (see [2, Chapter 4]).

We develop a set of limit definitions, viscosity solution characterizations, auxiliary lemmas and a core convergence theorem to analyze the penalized HJB variational inequality. The mathematical constructions presented below hold over the full feasible domain and all admissible parameters, and the derived theoretical results apply uniformly to both Black-Scholes constant-volatility and Heston stochastic-volatility models without restrictive parameter constraints.

**Definition 0.1.** *Suppose the operator* $ℱ:{\mathbb{R}}^{3}\times \mathbb{R}\times [0,T]\times {\mathbb{R}}_{+}\to \mathbb{R}$ *is locally Lipschitz continuous, and let* $\hat{V}:[0,T]\times {\mathbb{R}}_{+}\to \mathbb{R}$ *be locally bounded. For arbitrary constants* ρ>0,h>0*, we use B(z,r) to denote the open neighborhood centered at z with radius r. For any state* $(t,x)\in [0,T]\times {\mathbb{R}}_{+}$*, the upper semi-continuous envelope* $\overset{―}{ℱ}(t,x)$ *and lower semi-continuous envelope* $\underset{―}{ℱ}(t,x)$ *of* ℱ *are defined as*


$$\begin{array}{cc} & \overset{―}{ℱ}(t,x)\equiv \underset{\begin{array}{c} \tilde{t}\to t\\ \tilde{x}\to x\\ \tilde{t}\in B(t,\rho )\\ \tilde{x}\in B(x,h) \end{array}}{\lim\sup}ℱ({\hat{V}}_{xx}(\tilde{t},\tilde{x}),{\hat{V}}_{x}(\tilde{t},\tilde{x}),{\hat{V}}_{t}(\tilde{t},\tilde{x}),\hat{V}(\tilde{t},\tilde{x}),\tilde{t},\tilde{x}),\\ & \underset{―}{ℱ}(t,x)\equiv \underset{\begin{array}{c} \tilde{t}\to t\\ \tilde{x}\to x\\ \tilde{t}\in B(t,\rho )\\ \tilde{x}\in B(x,h) \end{array}}{\lim\inf}ℱ({\hat{V}}_{xx}(\tilde{t},\tilde{x}),{\hat{V}}_{x}(\tilde{t},\tilde{x}),{\hat{V}}_{t}(\tilde{t},\tilde{x}),\hat{V}(\tilde{t},\tilde{x}),\tilde{t},\tilde{x}). \end{array}$$

**Definition 0.2.** *Let* λ>0 *denote the positive penalty parameter, and* $\hat{V}:[0,T]\times {\mathbb{R}}_{+}\to \mathbb{R}$ *be locally bounded. For any state* $(t,x)\in [0,T]\times {\mathbb{R}}_{+}$*, the upper weak limit* ${\hat{V}}^{*}(t,x)$ *and lower weak limit* ${\hat{V}}_{*}(t,x)$ *of* $\hat{V}(t,x)$ *are respectively defined by*


$$\begin{array}{c} {\hat{V}}^{*}(t,x)\equiv \underset{\begin{array}{c} \tilde{t}\to t\\ \tilde{x}\to x\\ \tilde{t}\in B(t,1/\lambda )\\ \tilde{x}\in B(x,1/\lambda )\\ \lambda \to \infty \end{array}}{lim sup}\hat{V}(\tilde{t},\tilde{x})\;\text{and}\;{\hat{V}}_{*}(t,x)\equiv \underset{\begin{array}{c} \tilde{t}\to t\\ \tilde{x}\to x\\ \tilde{t}\in B(t,1/\lambda )\\ \tilde{x}\in B(x,1/\lambda )\\ \lambda \to \infty \end{array}}{lim inf}\hat{V}(\tilde{t},\tilde{x}), \end{array}$$

*where the neighborhood notation* B(·,·) *follows Definition 0.1.*

**Definition 0.3.** *Let* $\overset{―}{\Omega }=[0,T]\times {\mathbb{R}}_{+}$ *be the closed state domain, and* $\hat{V}:\overset{―}{\Omega }\to \mathbb{R}$ *be locally bounded.*

*(i) If for all test functions* $\varphi \in C^{1,2}(\overset{―}{\Omega })$*, and every point* $(\overset{―}{t},\overset{―}{x})\in \overset{―}{\Omega }$ *such that* ${\hat{V}}^{*}-\varphi$ *attains a strict local maximum at* $\left(\overset{―}{t},\overset{―}{x}\right)$*, the inequality*


$$\begin{array}{c} \underset{―}{ℱ}\left(\varphi_{xx}(\overset{―}{t},\overset{―}{x}),\varphi_{x}(\overset{―}{t},\overset{―}{x}),\varphi_{t}(\overset{―}{t},\overset{―}{x}),{\hat{V}}^{*},\overset{―}{x},\overset{―}{t}\right)\leq 0 \end{array}$$

*holds, then* $\hat{V}$ *is called a viscosity* subsolution of (12).

*(ii) If for all test functions* $\varphi \in C^{1,2}(\overset{―}{\Omega })$*, and every point* $(\underset{―}{t},\underset{―}{x})\in \overset{―}{\Omega }$ *such that* ${\hat{V}}_{*}-\varphi$ *attains a strict local minimum at* $\left(\underset{―}{t},\underset{―}{x}\right)$*, the inequality*


$$\begin{array}{c} \overset{―}{ℱ}\left(\varphi_{xx}(\underset{―}{t},\underset{―}{x}),\varphi_{x}(\underset{―}{t},\underset{―}{x}),\varphi_{t}(\underset{―}{t},\underset{―}{x}),{\hat{V}}_{*},\underset{―}{x},\underset{―}{t}\right)\geq 0 \end{array}$$

*holds, then* $\hat{V}$ *is called a viscosity supersolution of* (12).

*(iii) If* $\hat{V}$ *is both a viscosity subsolution and a viscosity supersolution of* (12)*, we term* $\hat{V}$ *a viscosity solution of* (12).

**Lemma 0.1.** *Let* λ>0 *be an arbitrary penalty parameter,* $V(t,x):\overset{―}{\Omega }\to \mathbb{R}$ *denote the unique viscosity solution of penalized HJB* equation (10) (w*hose existence and uniqueness are guaranteed by the comparison principle; see [2, Chapter 4]), and define* $\overset{―}{V}(t,x)=V^{*}(t,x)$*,* $\underset{―}{V}(t,x)=V_{*}(t,x)$ *as the upper and lower weak limits of V(t, x) via Definition 0.2. The following properties hold for all* $(t,x)\in \overset{―}{\Omega }$*:*

***(i)*** $\overset{―}{V}(t,x)$ *is upper semi-continuous on* $\overset{―}{\Omega }$ *and* $\underset{―}{V}(t,x)$ *is lower semi-continuous on* $\overset{―}{\Omega }$.
***(ii)*** *Suppose* $\overset{―}{V}$ *is upper semi-continuous (resp.* $\underset{―}{V}$ *lower semi-continuous), and* $(\overset{―}{t},\overset{―}{x})\in \overset{―}{\Omega }$ *is a strict local maximizer of* $\overset{―}{V}-\varphi$ *(resp. strict local minimizer of* $\underset{―}{V}-\varphi$*) for some test function* $\varphi \in C^{1,2}(\overset{―}{\Omega })$*. Then there exists a subsequence* $\{(t_{\lambda },x_{\lambda })\}\subset \overset{―}{\Omega }$ *satisfying* $\left(t_{\lambda },x_{\lambda })\to (\overset{―}{t},\overset{―}{x}\right)$ *and* $V(t_{\lambda },x_{\lambda })\to \overset{―}{V}(\overset{―}{t},\overset{―}{x})$ *(resp.* $\underset{―}{V}(\overset{―}{t},\overset{―}{x})$*) as* λ→∞*. Moreover,* $\left(t_{\lambda },x_{\lambda }\right)$ *is a local maximizer (resp. local minimizer) of* V−φ *for each* λ>0.

**Theorem 0.1.** *Fix any penalty parameter* λ>0*. Let* $V(t,x):\overset{―}{\Omega }\to \mathbb{R}$ *be the unique viscosity solution of penalized HJB* equation (43), and *define* $\overset{―}{V}(t,x),\underset{―}{V}(t,x)$ *as the upper and lower weak limits of V(t,x) according to Definition 0.2. Then* $\overset{―}{V}(t,x)$ *is a viscosity subsolution and* $\underset{―}{V}(t,x)$ *is a viscosity supersolution of the original HJB variational inequality* (5)*.*

*Proof.* Throughout this section, we work under the standard compactness assumption that the state domain is compact, or more generally, that the relevant sequences admit convergent subsequences; see [2, Chapter 4]. This assumption is standard in the viscosity solution framework for convergence proofs and is satisfied under our model settings.

We first establish that $\overset{―}{V}(t,x)$ is a viscosity subsolution of (5). To this end, consider any test function $\psi (t,x)\in C^{1,2}([0,T]\times \overset{―}{\Omega })$, and assume that $\left(\bar{t},\bar{x}\right)$ is a strict local maximizer of $\overset{―}{V}(t,x)-\psi (t,x)$. It then suffices to verify that the following inequality holds:


$$\begin{array}{c} \underset{―}{ℱ}(\psi_{xx}(\overset{―}{t},\overset{―}{x}),\psi_{x}(\overset{―}{t},\overset{―}{x}),\psi_{t}(\overset{―}{t},\overset{―}{x}),\overset{―}{V}(\overset{―}{t},\overset{―}{x}),\overset{―}{t},\overset{―}{x})\leq 0, \end{array}$$ (13)

that is,


$$\begin{array}{cc} \min\{ & \psi_{t}(\overset{―}{t},\overset{―}{x})-\underset{\pi }{\sup}\{\frac{1}{2}\sigma^{2}\pi^{2}{\overset{―}{x}}^{2}\cdot \psi_{xx}(\overset{―}{t},\overset{―}{x})\\ & +[\pi (\mu -r)+r]\overset{―}{x}\cdot \psi_{x}(\overset{―}{t},\overset{―}{x})-\beta \overset{―}{V}(\overset{―}{t},\overset{―}{x})\},\overset{―}{V}(\overset{―}{t},\overset{―}{x})-U(\overset{―}{x}-K)\}\leq 0. \end{array}$$ (14)

Suppose, to the contrary, that condition (13) (equivalently, (14)) is not satisfied, i.e.,


$$\begin{array}{c} \underset{―}{ℱ}(\psi_{xx}(\overset{―}{t},\overset{―}{x}),\psi_{x}(\overset{―}{t},\overset{―}{x}),\psi_{t}(\overset{―}{t},\overset{―}{x}),\overset{―}{V}(\overset{―}{t},\overset{―}{x}),\overset{―}{t},\overset{―}{x})>0. \end{array}$$ (15)

By Lemma 0.1, there exists a subsequence $\left\{(t_{\lambda },x_{\lambda })\right\}$ such that $\left(t_{\lambda },x_{\lambda })\to (\overset{―}{t},\overset{―}{x}\right)$ and $V(t_{\lambda },x_{\lambda })\to \overset{―}{V}(\overset{―}{t},\overset{―}{x})$ as λ→∞, where each $\left(t_{\lambda },x_{\lambda }\right)$ is a local maximizer of V(t,x)−φ(t,x). Since $\varphi \in C^{1,2}([0,T]\times \Omega )$ and condition (14) is continuous, there exists a neighborhood $B(\overset{―}{t},\overset{―}{x})\subset [0,T]\times \Omega$ such that the inequality holds for all $\left(t_{\lambda },x_{\lambda })\in B(\overset{―}{t},\overset{―}{x}\right)$,


$$\begin{array}{c} \underset{―}{ℱ}(\psi_{xx}(t_{\lambda },x_{\lambda }),\psi_{x}(t_{\lambda },x_{\lambda }),\psi_{t}(t_{\lambda },x_{\lambda }),\overset{―}{V}(t_{\lambda },x_{\lambda }),t_{\lambda },x_{\lambda })>0. \end{array}$$ (16)

By the definition of ℱ and (43), we can derive that


$$\begin{array}{c} \partial_{t}\psi (t_{\lambda },x_{\lambda })-ℋ\psi (t_{\lambda },x_{\lambda }))>0. \end{array}$$ (17)

Given that $\overset{―}{V}$ is a viscosity subsolution of (43) and each $\left(t_{\lambda },x_{\lambda }\right)$ is a strict local maximizer of $\overset{―}{V}(t,x)-\psi$, the definition of the viscosity solution for the penalized HJB equation (43) then yields:


$$\begin{array}{c} \partial_{t}\psi (\overset{―}{t},\overset{―}{x})-ℋ\psi (\overset{―}{t},\overset{―}{x})\leq 0. \end{array}$$ (18)

Inequalities (17) and (18) contradict one another. Consequently, the initial assumption (15) must be false. Therefore, at least one of the conditions (13) or (14) holds, which proves that $\overset{―}{V}$ is indeed a viscosity subsolution to (5).

We now prove that $\underset{―}{V}(t,x)$ is a viscosity supersolution to (5). It suffices to show that for any test function $\psi (t,x)\in C^{1,2}([0,T]\times \overset{―}{\Omega })$, if $\left(\overset{―}{t},\overset{―}{x}\right)$ is a strict local minimizer of $\underset{―}{V}(t,x)-\psi$, then the associated differential inequality holds.


$$\begin{array}{c} \overset{―}{ℱ}(\psi_{xx}(\overset{―}{t},\overset{―}{x}),\psi_{x}(\overset{―}{t},\overset{―}{x}),\psi_{t}(\overset{―}{t},\overset{―}{x}),\underset{―}{V}(\overset{―}{t},\overset{―}{x}),\overset{―}{t},\overset{―}{x})\geq 0, \end{array}$$ (19)

that is,


$$\begin{array}{cc} \min\{ & \psi_{t}(\overset{―}{t},\overset{―}{x})-\underset{\pi }{\sup}\{\frac{1}{2}\sigma^{2}\pi^{2}{\overset{―}{x}}^{2}\cdot \psi_{xx}(\overset{―}{t},\overset{―}{x})\\ & +[\pi (\mu -r)+r]\overset{―}{x}\cdot \psi_{x}(\overset{―}{t},\overset{―}{x})-\beta \underset{―}{V}(\overset{―}{t},\overset{―}{x})\},\underset{―}{V}(\overset{―}{t},\overset{―}{x})-U(\overset{―}{x}-K)\}\geq 0. \end{array}$$ (20)

Consider the following two cases:

Assume that


$$\begin{array}{c} \psi_{t}(\overset{―}{t},\overset{―}{x})-\underset{\pi }{\sup}\{\frac{1}{2}\sigma^{2}\pi^{2}{\overset{―}{x}}^{2}\cdot \psi_{xx}(\overset{―}{t},\overset{―}{x})+[\pi (\mu -r)+r]\overset{―}{x}\cdot \psi_{x}(\overset{―}{t},\overset{―}{x})-\beta \underset{―}{V}(\overset{―}{t},\overset{―}{x})\}<0. \end{array}$$ (21)

By Lemma 0.1, we may extract a subsequence $\left\{(t_{\lambda },x_{\lambda })\right\}$ satisfying $\left(t_{\lambda },x_{\lambda })\to (\overset{―}{t},\overset{―}{x}\right)$ and $V(t_{\lambda },x_{\lambda })\to \underset{―}{V}(\overset{―}{t},\overset{―}{x})$ as λ→∞, with each $\left(t_{\lambda },x_{\lambda }\right)$ being a local minimizer of V(t,x)−φ(t,x). Since $\varphi \in C^{1,2}([0,T]\times \Omega )$ and condition (21) is continuous, one can find a neighborhood $B(\overset{―}{t},\overset{―}{x})\subset [0,T]\times \Omega$ where the required inequality holds for all $\left(t_{\lambda },x_{\lambda })\in B(\overset{―}{t},\overset{―}{x}\right)$,


$$\begin{array}{cc} & \psi_{t}(t_{\lambda },x_{\lambda })-\underset{\pi }{\sup}\{\frac{1}{2}\sigma^{2}\pi^{2}x_{\lambda }^{2}\cdot \psi_{xx}(t_{\lambda },x_{\lambda })\\ & +[\pi (\mu -r)+r]x_{\lambda }\cdot \psi_{x}(t_{\lambda },x_{\lambda })-\beta \underset{―}{V}(t_{\lambda },x_{\lambda })\}<0. \end{array}$$

From this inequality, we deduce that for any λ>0,


$$\begin{array}{cc} \psi_{t}(t_{\lambda },x_{\lambda })-\underset{\pi }{\sup}\{ & \frac{1}{2}\sigma^{2}\pi^{2}x_{\lambda }^{2}\cdot \psi_{xx}(t_{\lambda },x_{\lambda })\\ & +[\pi (\mu -r)+r]x_{\lambda }\cdot \psi_{x}(t_{\lambda },x_{\lambda })-\beta \underset{―}{V}(t_{\lambda },x_{\lambda })\}\\ & -\lambda (U(x_{\lambda }-K)-\underset{―}{V}(t_{\lambda },x_{\lambda }){)}^{+}<0, \end{array}$$

that is,


$$\begin{array}{c} \partial_{t}\psi (t_{\lambda },x_{\lambda })-ℋ\psi (t_{\lambda },x_{\lambda })<0. \end{array}$$ (22)

Under the hypotheses that $\underset{―}{V}$ is a viscosity supersolution to (43) and each $\left(t_{\lambda },x_{\lambda }\right)$ is a strict local minimizer of $\underset{―}{V}(t,x)-\psi$ for any λ, the definition of viscosity solution for (43) gives


$$\begin{array}{c} \partial_{t}\psi (\overset{―}{t},\overset{―}{x})-ℋ\psi (\overset{―}{t},\overset{―}{x})\geq 0. \end{array}$$ (23)

This contradiction between (22) and (23) shows that the assumption (21) cannot hold.

Assume that


$$\begin{array}{cc} \psi_{t}(\overset{―}{t},\overset{―}{x})-\underset{\pi }{\sup}\{ & \frac{1}{2}\sigma^{2}\pi^{2}{\overset{―}{x}}^{2}\cdot \psi_{xx}(\overset{―}{t},\overset{―}{x})\\ & +[\pi (\mu -r)+r]\overset{―}{x}\cdot \psi_{x}(\overset{―}{t},\overset{―}{x})-\beta \underset{―}{V}(\overset{―}{t},\overset{―}{x})\}\\ \equiv & \zeta \geq 0, \end{array}$$ (24)

and


$$\begin{array}{c} \underset{―}{V}(\overset{―}{t},\overset{―}{x})-U(\bar{x}-K)\equiv \eta <0. \end{array}$$ (25)

By Lemma 0.1, we can select a subsequence $\left\{(t_{\lambda },x_{\lambda })\right\}$ such that $\left(t_{\lambda },x_{\lambda })\to (\overset{―}{t},\overset{―}{x}\right)$ and $V(t_{\lambda },x_{\lambda })\to \underset{―}{V}(\overset{―}{t},\overset{―}{x})$ as λ→∞, with each $\left(t_{\lambda },x_{\lambda }\right)$ acting as a local minimizer of V(t,x)−φ(t,x). Since $\varphi \in C^{1,2}([0,T]\times \Omega )$, and in view of the continuity of (24) and (25), one may choose a neighborhood $B(\overset{―}{t},\overset{―}{x})\subset [0,T]\times \Omega$ where the preceding inequality holds for all $\left(t_{\lambda },x_{\lambda })\in B(\overset{―}{t},\overset{―}{x}\right)$,


$$\begin{array}{cc} \zeta -\frac{\left|\eta \right|}{2}\leq & \psi_{t}(t_{\lambda },x_{\lambda })-\underset{\pi }{\sup}\{\frac{1}{2}\sigma^{2}\pi^{2}x_{\lambda }^{2}\cdot \psi_{xx}(t_{\lambda },x_{\lambda })\\ & +[\pi (\mu -r)+r]x_{\lambda }\cdot \psi_{x}(t_{\lambda },x_{\lambda })-\beta \underset{―}{V}(t_{\lambda },x_{\lambda })\}\\ \leq & \zeta +\frac{\left|\eta \right|}{2}. \end{array}$$ (26)

and


$$\begin{array}{c} \eta -\frac{\left|\eta \right|}{2}\leq \underset{―}{V}(t_{\lambda },x_{\lambda })-U(x_{\lambda }-K)\leq \eta +\frac{\left|\eta \right|}{2}. \end{array}$$ (27)

For λ>(2ζ+|η|)/|η|, we can combine (26) with (27) to obtain


$$\begin{array}{cc} & \psi_{t}(t_{\lambda },x_{\lambda })-\underset{\pi }{\sup}\{\frac{1}{2}\sigma^{2}\pi^{2}x_{\lambda }^{2}\cdot \psi_{xx}(t_{\lambda },x_{\lambda })\\ & +[\pi (\mu -r)+r]x_{k(t)}\cdot \psi_{x}(t_{\lambda },x_{\lambda })-\beta \underset{―}{V}(t_{\lambda },x_{\lambda })\}\\ & -\lambda (U(x_{\lambda }-K)-\underset{―}{V}(t_{\lambda },x_{\lambda }){)}^{+}\\ < & \zeta +\frac{\left|\eta \right|}{2}+\frac{2\zeta +|\eta |}{\left|\eta \right|}(\eta +\frac{\left|\eta \right|}{2})=\zeta -\frac{\eta }{2}-\frac{2\zeta -\eta }{\eta }(\eta -\frac{\eta }{2})=0, \end{array}$$

that is,


$$\begin{array}{c} \partial_{t}\psi (t_{\lambda },x_{\lambda })-ℋ\psi (t_{\lambda },x_{\lambda })<0. \end{array}$$ (28)

Since $\underset{―}{V}$ is assumed to be a viscosity supersolution to the penalized HJB equation (43), and each $\left(t_{\lambda },x_{\lambda }\right)$ is a strict local minimizer of $\underset{―}{V}(t,x)-\psi$ for all λ, the definition of viscosity supersolution for (43) yields that


$$\begin{array}{c} \partial_{t}\psi (\overset{―}{t},\overset{―}{x})-ℋ\psi (\overset{―}{t},\overset{―}{x})\geq 0. \end{array}$$ (29)

A contradiction arises from (28) and (29); accordingly, assumptions (24) and (25) cannot hold.

Combining the two cases considered above, we infer that $\underset{―}{V}$ is a viscosity supersolution to (5), i.e., either (19) or (20) is satisfied. □

### GLL spectral discretization with policy iteration

In this section, we develop an iterative GLL spectral method to solve the penalized HJB equation (43), based on the framework established in [22].

The Legendre polynomials $\left\{L_{n}(\xi ),\;n\geq 0\right\}$ form an orthogonal set on [−1,1] under a unit weight (see [26, Chapter 3] for their definitions and properties). Building upon them, the GLL quadrature rule is defined to include the two endpoints ξ=±1.


$$\begin{array}{cc} \int_{-1}^{1}f(\xi )d\xi \approx & {\bar{I}}_{n}^{\text{GLL}}\{f\}:=\sum_{k=0}^{n}\omega_{k}f(\xi_{k})\\ = & \omega_{0}f(-1)+\sum_{k=1}^{n-1}\omega_{k}f(\xi_{k})+\omega_{n}f(1), \end{array}$$ (30)

where $\{\xi_{k}{\}}_{k=0}^{n}$ denotes the roots of $L_{n+1}(\xi )-L_{n-1}(\xi )$; these nodes are distinct, with the endpoints −1 and 1 fixed and all other nodes lying strictly in (−1,1) (see [26, Chapter 3] for the standard proof of this property). The corresponding quadrature weights associated with these grid points are defined as


$$\begin{array}{c} \omega_{k}=\frac{2}{n(n+1)L_{n}^{2}(\xi_{k})},\;k=0,1,...,n. \end{array}$$ (31)

The GLL basis function ${\bar{\phi }}_{k}(\xi )$ is constructed in the form of a Lagrange interpolation polynomial and is defined by


$$\begin{array}{c} {\bar{\phi }}_{k}(\xi ):=\frac{L_{n+1}(\xi )-L_{n-1}(\xi )}{\left(2n+1)(\xi -\xi_{k})L_{n}(\xi_{k}\right)},\;k=0,1,...,n. \end{array}$$

These basis functions possess the interpolation property ${\bar{\phi }}_{k}(\xi_{l})=\delta_{kl}$ (see [27, 28]). Moreover, for 0≤k≤n, the first-order derivative of ${\bar{\phi }}_{l}(\xi )$ evaluated at the grid point $\xi_{k}$ is


$$\begin{array}{c} D_{kl}:=\frac{\partial {\bar{\phi }}_{l}(\xi_{k})}{\partial \xi }=\left\{ \begin{array}{cc} \frac{L_{n}(\xi_{k})}{\left(\xi_{k}-\xi_{l})L_{n}(\xi_{l}\right)},\; & \text{if }k\text{≠}l;\\ 0, & \text{if }k=l\text{≠}0,\;n;\\ -\frac{n(n+1)}{4}, & \text{if }k=l=0;\\ \frac{n(n+1)}{4}, & \text{if }k=l=n. \end{array}\right. \end{array}$$ (32)

The second-order derivative of the GLL basis function is also given by


$$\begin{array}{c} B_{kl}:=\frac{\partial^{2}{\bar{\phi }}_{l}(\xi_{k})}{\partial \xi^{2}}=\left\{ \begin{array}{cc} -\frac{2L_{n}(\xi_{k})}{\left(\xi_{k}-\xi_{l}{)}^{2}L_{n}(\xi_{l}\right)}, & \text{if }k\text{≠}l,\;k\text{≠}0,\;n;\\ (-1{)}^{n}\frac{n(n+1)(\xi_{k}-\xi_{l})-4}{2(\xi_{k}-\xi_{l}{)}^{2}L_{n}(\xi_{l})}, & \text{if }k\text{≠}l,\;k=0;\\ \frac{n(n+1)(1-\xi_{l})-4}{2(1-\xi_{l}{)}^{2}L_{n}(\xi_{l})}, & \text{if }k\text{≠}l,\;k=n;\\ -\frac{n(n+1)}{3(1-\xi_{k}^{2})}, & \text{if }k=l\text{≠}0,\;n;\\ \frac{\left(n-1)n(n+1)(n+2\right)}{24}, & \text{if }k=l=0,\;n. \end{array}\right. \end{array}$$ (33)

The computational domain Ω is mapped onto the reference interval $\bar{\Omega }=[-1,1]$ via


$$\begin{array}{c} \frac{x-K}{x_{\max}-K}=\frac{\xi +1}{2}\;\text{or}\;x=J\xi +\frac{x_{\max}+K}{2},\;\\ J=\frac{x_{\max}-K}{2},\;\xi \in \bar{\Omega },\;x\in \Omega . \end{array}$$ (34)

On a general interval $\left(K,x_{\max}\right)$, the GLL quadrature rule (30) takes the form


$$\begin{array}{cc} \int_{K}^{x_{\max}}f(x)dx & =J\int_{-1}^{1}f\left(J\xi +\frac{x_{\max}+K}{2}\right)d\xi \\ & :=J\int_{-1}^{1}\bar{f}\left(\xi \right)d\xi \approx J{\bar{I}}_{n}^{\text{GLL}}\{\bar{f}\}\\ & :=I_{n,(K,x_{\max})}^{\text{GLL}}\{f\}. \end{array}$$ (35)

Define polynomial space


$$\begin{array}{c} {𝕍}_{n}(\Omega ):=\text{span}\left\{\phi_{0}(x),\phi_{1}(x),\cdots ,\phi_{n}(x)\right\}, \end{array}$$

where $\phi_{l}$ is respectively the transformation of the GLL basis ${\bar{\phi }}_{l}$ into Ω using (34), namely,


$$\begin{array}{c} {\bar{\phi }}_{l}(\xi )={\bar{\phi }}_{l}\left(\frac{2(x-K)}{x_{\max}-K}-1\right)=:\phi_{l}(x),\;l=0,1,...,n. \end{array}$$

Define inner product


$$\begin{array}{c} (\nu ,\mu ):=\int_{\Omega }\nu (x)\mu (x)dx. \end{array}$$

Then using the expansion


$$\begin{array}{c} V(t,x)\approx \sum_{l=0}^{n}V_{l}(t)\phi_{l}(x), \end{array}$$

to replace *V* in (43) and taking inner product by $\phi_{k}(x)$ for k=1,...,n−1 give that


$$\begin{array}{cc} 0 & =\sum_{l=1}^{n}V_{l}^{\prime }(t)(\phi_{l},\phi_{k})-\{\sum_{l=1}^{n}V_{l}(t)[\frac{1}{2}\sigma^{2}(\pi_{*}^{2}(t,x)x^{2}\phi_{l}^{\prime \prime },\phi_{k})\\ & +[(\mu -r)+r](\pi_{*}(t,x)x\phi_{l}^{\prime },\phi_{k})-\beta (\phi_{l},\phi_{k})]\}\\ & \;-\lambda ((U(x-K)-\sum_{l=0}^{n}V_{l}(t)\phi_{l}{)}^{+},\phi_{k}),\;k=1,...,n-1, \end{array}$$ (36)

where


$$\begin{array}{cc} \pi_{*}(t,x) & \in \underset{\pi }{\text{arg}\sup}{ℒ}^{\pi }V\\ & =\underset{\pi }{\text{arg}\sup}\left[\frac{1}{2}\sigma^{2}\pi^{2}x^{2}\cdot V_{xx}(t,x)+[\pi (\mu -r)+r]x\cdot V_{x}(t,x)-\beta V(t,x)\right]. \end{array}$$

Define temporal grids $t_{i}=i\Delta t,\;i=0,1,...,m$ with $\Delta t=\frac{T}{m+1}$, and let $V_{l}^{i}\approx V_{l}(t_{i})$. Then with the time discretization of (46), the fully discretized scheme for (43) is defined as


$$\begin{array}{cc} & \sum_{l=0}^{n}(V_{l}^{i+1}-V_{l}^{i})(\phi_{l},\phi_{k})\\ = & \Delta t\{\sum_{l=1}^{n}[\frac{1}{2}\sigma^{2}(\pi_{*}^{2}(t,x)x^{2}\phi_{l}^{\prime \prime },\phi_{k})+[(\mu -r)+r](\pi_{*}(t,x)x\phi_{l}^{\prime },\phi_{k})-\beta (\phi_{l},\phi_{k})]\\ & (\eta_{k,l}V_{l}^{i}+(1-\eta_{k,l})V_{l}^{i+1})+\lambda ((U(x-K)-\sum_{l=0}^{n}V_{l}^{i}\phi_{l}{)}^{+},\phi_{k})\}, \end{array}$$ (37)

k=1,...,n−1, where


$$\begin{array}{c} V_{0}^{i}=0\;\text{and}\;V_{n}^{i}=\Psi (t_{i},x_{\max}), \end{array}$$

and $\eta_{k,l}=0$ or 1 will be specified later.

We adopt GLL quadrature to evaluate the integrals in (47), which preserves spectral accuracy. The resulting stiffness, advection, and mass matrices are given as follows:


$$\begin{array}{cc} S_{kl} & =\int_{\Omega }\frac{\partial^{2}\phi_{l}(x)}{\partial x^{2}}\phi_{k}(x)dx=\frac{1}{J}\int_{-1}^{1}\frac{\partial^{2}{\bar{\phi }}_{l}(\xi )}{\partial \xi }{\bar{\phi }}_{k}(\xi )d\xi \approx \frac{1}{J}\sum_{s=0}^{n}w_{s}B_{sl}\delta_{sk}=\frac{1}{J}\omega_{k}B_{kl},\\ A_{kl} & =\int_{\Omega }\frac{\partial \phi_{l}(x)}{\partial x}\phi_{k}(x)dx=\int_{-1}^{1}\frac{\partial {\bar{\phi }}_{l}(\xi )}{\partial \xi }{\bar{\phi }}_{k}(\xi )d\xi \approx \sum_{s=0}^{n}w_{s}D_{sl}\delta_{sk}=\omega_{k}D_{kl},\\ M_{kl} & =\int_{\Omega }\phi_{l}(x)\phi_{k}(x)dx=J\int_{-1}^{1}{\bar{\phi }}_{l}(\xi ){\bar{\phi }}_{k}(\xi )d\xi \approx J\sum_{s=0}^{n}w_{s}\phi_{l}(\xi_{s})\phi_{k}(\xi_{s})=J\omega_{l}\delta_{kl}. \end{array}$$

Applying GLL quadrature to (47) yields the following discrete system:


$$\begin{array}{cc} & V_{k}^{i+1}-V_{k}^{i}\\ & \;=\frac{\Delta t}{J\omega_{k}}\{\sum_{l=0}^{n}[\frac{1}{2}\sigma^{2}\pi_{k}^{2}x_{k}^{2}S_{kl}+[\pi_{k}(\mu -r)+r]x_{k}A_{kl}-\beta M_{kl}]\\ & \;\left(\eta_{k,l}V_{l}^{i}+(1-\eta_{k,l})V_{l}^{i+1}\right)+\lambda J\omega_{k}(U(x_{k}-K)-V_{k}^{i}{)}^{+}\}, \end{array}$$ (38)

where $\pi_{k}=\pi_{*}(t_{i+1},x_{k})$.

For analytical convenience, we transform the system (48) into the following matrix-vector notation:


$$\begin{array}{cc} & {𝐕}^{i}=[V_{0}^{i},V_{1}^{i},\cdots ,V_{n}^{i}{]}^{⊤},\\ & [{𝐕}^{i}{]}_{1:n-1}=[V_{1}^{i},...,V_{n-1}^{i}{]}^{⊤},\\ & 𝐏{𝐕}^{i}=[\lambda (U(x_{0}-K)-V_{0}^{i}{)}^{+},\lambda (U(x_{1}-K)-V_{1}^{i}{)}^{+},\cdots ,\lambda (U(x_{n}-K)-V_{n}^{i}{)}^{+}{]}^{⊤}. \end{array}$$

Define matrices


$$\begin{array}{c} {}[{𝐄}_{i}^{\pi }{]}_{k,l}=\frac{\eta_{k,l}}{J\omega_{k}}{\left[{𝐐}_{i}^{\pi }\right]}_{k,l},\;[{𝐈}_{i}^{\pi }{]}_{k,l}=\frac{1-\eta_{k,l}}{J\omega_{k}}{\left[{𝐐}_{i}^{\pi }\right]}_{k,l}, \end{array}$$ (39)

where k=1,...,n−1, l=0,...,n, where $\omega_{k}$ and J given by (31) and (34) respectively are positive,


$$\begin{array}{c} {\left[{𝐐}_{i}^{\pi }\right]}_{k,l}:=\frac{1}{2}\sigma^{2}\pi_{k}^{2}x_{k}^{2}S_{kl}+[\pi_{k}(\mu -r)+r]x_{k}A_{kl}-\beta M_{kl}, \end{array}$$

and


$$\begin{array}{cc} \eta_{k,l}= & \left\{ \begin{array}{c} 0,\;{\left[{𝐐}_{i}^{\pi }\right]}_{k,l}>0,\\ 1,\;{\left[{𝐐}_{i}^{\pi }\right]}_{k,l}\leq 0. \end{array}\right. \end{array}$$

Then (48) can be written as


$$\begin{array}{c} Id[{𝐕}^{i+1}{]}_{1:n-1}-Id[{𝐕}^{i}{]}_{1:n-1}-\Delta t\left\{{𝐄}_{i}^{\pi }{𝐕}^{i}+{𝐈}_{i}^{\pi }{𝐕}^{i+1}+[𝐏{𝐕}^{i}{]}_{1:n-1}\right\}=0. \end{array}$$ (40)

To implement the scheme (40), we employ the following policy iteration algorithm.

**Algorithm 1 Iteration policy for solving (40)**

1:  **for** i=0,1,...,m−1 **do**

2:   let $[{𝐕}^{i+1,(0)}{]}_{1:n-1}=[{𝐕}^{i}{]}_{1:n-1}$,

3:   **for** s=0,1,2,... **do**

4:    solve

$\begin{array}{cc} & Id[{𝐕}^{i+1,(s+1)}{]}_{1:n-1}-Id[{𝐕}^{i}{]}_{1:n-1}\\ - & \Delta t\left\{{𝐄}_{i}^{\pi^{\left(s\right)}}{𝐕}^{i}+{𝐈}_{i}^{\pi^{\left(s\right)}}{𝐕}^{i+1,(s+1)}+[𝐏{𝐕}^{i}{]}_{1:n-1}\right\}=0 \end{array}$

5:    where $\pi^{\left(s\right)}\in \underset{\pi }{\text{arg}\sup}{ℒ}^{\pi }{𝐕}^{i+1,(s)}$,

6:    **if** $\left[\max\left(\frac{\left|{𝐕}^{i+1,(s+1)}-{𝐕}^{i+1,(s)}\right|}{\max(1,|{𝐕}^{i+1,(s+1)}|)}\right)<\text{tolerance}\right]$,

7:     ley ${𝐕}^{i+1}={𝐕}^{i+1,(s+1)}$, then quit.

8:    **end if**

9:   **end for**

10:  **end for**

### Extended model: Heston stochastic volatility framework

The Black-Scholes (BS) model in the model formulation section under the Black-Scholes framework is established under constant volatility. To further enrich the model framework and introduce time-varying volatility characteristics, this section extends the baseline model to the Heston stochastic volatility framework. This extension adds instantaneous variance as a new state variable to construct a two-dimensional optimal stopping system. Consistent with the modeling framework in the BS model setup described above, this paper retains the same probability space, market assumptions, utility function, admissible strategy set and time transformation rule. Only the asset price dynamics, wealth process and infinitesimal generator are updated to adapt to the stochastic volatility environment. We introduce two correlated Brownian motions $W_{\tau }^{s}$ and $W_{\tau }^{v}$ defined on the pre-specified probability space, with correlation coefficient ρ∈(−1,1). The instantaneous variance $v_{\tau }$ follows a mean-reverting CIR process, and the joint dynamic equations of asset price and variance are formulated as


$$\begin{array}{c} \left\{ \begin{array}{c} dS_{\tau }=\mu S_{\tau }d\tau +\sqrt{v_{\tau }}S_{\tau }dW_{\tau }^{s},\\ dv_{\tau }=\kappa (\theta -v_{\tau })d\tau +\zeta \sqrt{v_{\tau }}dW_{\tau }^{v}, \end{array}\right. \end{array}$$

where κ represents the mean-reversion speed of variance, θ denotes the long-run equilibrium variance level, and ζ is the volatility-of-volatility parameter. The Feller condition $2\kappa \theta \geq \zeta^{2}$ is imposed to guarantee the non-negativity of the variance process. All other fundamental market parameters remain consistent with the baseline model. The updated wealth process under the stochastic volatility setting is expressed as


$$\begin{array}{c} dX_{\tau }=(rX_{\tau }+\pi_{\tau }(\mu -r)X_{\tau })d\tau +\pi_{\tau }X_{\tau }\sqrt{v_{\tau }}dW_{\tau }^{s}. \end{array}$$

Consistent with the model settings in the BS model setup described above, we adopt the same investment strategy constraint, discount factor, wealth threshold and utility function. Correspondingly, we define the forward value function for the extended two-dimensional state system composed of wealth *x* and instantaneous variance *v* as


$$\begin{array}{c} \tilde{V}(\tau ,x,v)=\underset{\pi \in 𝒜,\varsigma }{\sup}𝔼\left[e^{-\beta \varsigma }U(X_{\varsigma }-K)\;|\;X_{\tau }=x,\;v_{\tau }=v\right], \end{array}$$

The value function follows the same optimization objective and wealth constraint as the baseline model. Based on the dynamic programming principle applied in the BS model setup, the value function satisfies the variational inequality with the updated Heston infinitesimal generator. We employ the identical time transformation t=T−τ and define the backward value function $\hat{V}(t,x,v)=\tilde{V}(T-t,x,v)$. The backward variational inequality maintains the same min-max structure as the baseline framework, and the Heston infinitesimal generator is given by


$$\begin{array}{cc} {ℒ}_{\text{Heston}}^{\pi }\hat{V}= & (rx+\pi x(\mu -r)){\hat{V}}_{x}+\kappa (\theta -v){\hat{V}}_{v}+\frac{1}{2}\pi^{2}x^{2}v{\hat{V}}_{xx}+\frac{1}{2}\zeta^{2}v{\hat{V}}_{vv}+\rho \pi x\zeta v{\hat{V}}_{xv}. \end{array}$$ (41)

Following the unified economic specification criterion of the baseline model, we define the terminal and boundary conditions for the Heston model over the feasible domain of time, wealth and variance:


$$\begin{array}{c} \left\{ \begin{array}{cc} \hat{V}(0,x,v)=U(x-K), & (x,v)\in \Omega \times \Omega^{\prime }:=[v_{\min},v_{\max}],\\ \hat{V}(t,K,v)=0, & (t,v)\in [0,T]\times \Omega^{\prime },\\ \hat{V}(t,x_{\text{max}},v)=\Psi (t,x_{\text{max}}), & (t,v)\in [0,T]\times \Omega^{\prime }. \end{array}\right. \end{array}$$ (42)

The terminal condition specifies the utility payoff at maturity determined by excess wealth, while the lower and upper boundary conditions follow the identical constraint rules of the baseline model, which are valid for all feasible variance values within the defined interval.

Following the penalty equation derivation framework established in the penalty reformulation and convergence analysis section, we can directly obtain the penalty HJB equation under the Heston model,


$$\begin{array}{c} V_{t}(t,x,v)-\underset{\pi }{\sup}{ℒ}_{\text{Heston}}^{\pi }V-\lambda (U(x-K)-V(t,x,v){)}^{+}=0, \end{array}$$ (43)

on $[0,T]\times \Omega \times \Omega^{\prime }$, and the terminal and boundary conditions are given by


$$\begin{array}{cc} & V(0,x,v)=U(x-K),\;(x,v)\in \Omega \times \Omega^{\prime }\\ & V(t,K,v)=0,\;(t,v)\in [0,T]\times \Omega^{\prime },\\ & V(t,x_{\max},v)=\Psi (t,x_{\max},v)\;(t,v)\in [0,T]\times \Omega^{\prime }. \end{array}$$ (44)

The convergence of the viscosity solution of the resulting penalized equation to that of the original HJB variational inequality is consistent with the BS model. Thus, the relevant proof is not repeated here.

Now we develop an iterative GLL spectral method to solve the penalized HJB equation (43). The computational domain $\Omega^{\prime }$ is mapped onto the reference interval $\bar{\Omega }=[-1,1]$ via


$$\begin{array}{c} \frac{v-v_{\min}}{v_{\max}-v_{\min}}=\frac{\xi +1}{2}\;\text{or}\;v=J^{\prime }\xi +\frac{v_{\max}+v_{\min}}{2},\;\\ J^{\prime }=\frac{v_{\max}-v_{\min}}{2},\;\xi \in \bar{\Omega },\;v\in \Omega^{\prime }, \end{array}$$ (45)

Define polynomial space


$$\begin{array}{c} {𝕍}_{n_{1},n_{2}}(\Omega \times \Omega^{\prime }):=\text{span}\left\{(\phi_{0}(x),\phi_{1}(x),\cdots ,\phi_{n_{1}}(x))\times (\psi_{0}(v),\psi_{1}(v),\cdots ,\psi_{n_{2}}(v))\right\}, \end{array}$$

where $\psi_{k}$ is respectively the transformation of the GLL basis ${\bar{\phi }}_{k}$ into $\Omega^{\prime }$ using (45), namely,


$$\begin{array}{c} {\bar{\phi }}_{k}(\xi )={\bar{\phi }}_{k}\left(\frac{2(v-v_{\min})}{v_{\max}-v_{\min}}-1\right)=:\psi_{k}(v),\;k=0,1,...,n_{2}. \end{array}$$

Then using the expansion


$$\begin{array}{c} V(t,x,v)\approx \sum_{k=0}^{n_{2}}\sum_{l=0}^{n_{1}}V_{lk}(t)\phi_{l}(x)\psi_{k}(v), \end{array}$$

to replace *V* in (43) and taking inner product by $\phi_{p}(x)$ and $\psi_{q}(v)$ for $p=1,...,n_{1}-1$ and $q=0,...,n_{2}$ give that


$$\begin{array}{cc} 0= & \sum_{k=0}^{n_{2}}\sum_{l=0}^{n_{1}}V_{lk}^{\prime }(t)(\phi_{l}(x),\phi_{p}(x))(\psi_{k}(v),\psi_{q}(v))\\ & -\{\sum_{l=0}^{n_{1}}\sum_{k=0}^{n_{2}}V_{lk}(t)[r(x\phi_{l}^{\prime }(x),\phi_{p}(x))(\psi_{k}(v),\psi_{q}(v))\\ & +A((x\pi_{*}(t,x,v)\phi_{l}^{\prime }(x),\phi_{p}(x))v\psi_{k}(v),\psi_{q}(v))\\ & +\kappa \theta (\phi_{l}(x),\phi_{p}(x))(\psi_{k}^{\prime }(v),\psi_{q}(v))-\kappa (\phi_{l}(x),\phi_{p}(x))(v\psi_{k}^{\prime }(v),\psi_{q}(v))\\ & +\frac{1}{2}((\pi_{*}^{2}(t,x,v)x^{2}\phi_{l}^{\prime \prime }(x),\phi_{p}(x))v\psi_{k}(v),\psi_{q}(v))+\frac{1}{2}\zeta^{2}(\phi_{l}(x),\phi_{p}(x))(v\psi_{k}^{\prime \prime }(v),\psi_{j}(v))\\ & +\rho \zeta ((x\pi_{*}(t,x,v)\phi_{l}^{\prime }(x),\phi_{p}(x))v\psi_{k}^{\prime }(v),\psi_{q}(v))-\beta (\phi_{l}(x),\phi_{p}(x))(\psi_{k}(v),\psi_{q}(v))]\}\\ & -\lambda ((U(x-K)-\sum_{k=0}^{n_{2}}\sum_{l=0}^{n_{1}}V_{lk}(t)\phi_{l}(x)\psi_{k}(v){)}^{+},\phi_{p}(x)),\psi_{q}(v)), \end{array}$$ (46)

where


$$\begin{array}{cc} \pi_{*}(t,x,v) & \in \underset{\pi }{\text{arg}\sup}{ℒ}_{\text{Heston}}^{\pi }V\\ & =\underset{\pi }{\text{arg}\sup}[(rx+\pi xAv)V_{x}+\kappa (\theta -v)V_{v}+\frac{1}{2}\pi^{2}x^{2}vV_{xx}+\frac{1}{2}\zeta^{2}vV_{vv}\\ & +\rho \pi x\zeta vV_{xv}-\beta V]. \end{array}$$

Define temporal grids $t_{i}=i\Delta t,\;i=0,1,...,m$ with $\Delta t=\frac{T}{m+1}$, and let $V_{lk}^{i}\approx V_{lk}(t_{i})$. Then with the time discretization of (46), the fully discretized scheme for (43) is defined as


$$\begin{array}{cc} & \sum_{k=0}^{n_{2}}\sum_{l=0}^{n_{1}}(V_{lk}^{i+1}-V_{lk}^{i})(\phi_{l}(x),\phi_{p}(x))(\psi_{k}(v),\psi_{q}(v))\\ = & \Delta t\{\sum_{l=0}^{n_{1}}\sum_{k=0}^{n_{2}}(\eta_{lk}V_{lk}^{i}+(1-\eta_{lk})V_{lk}^{i+1})[r(x\phi_{l}^{\prime }(x),\phi_{p}(x))(\psi_{k}(v),\psi_{q}(v))\\ & +A((x\pi_{*}(t,x,v)\phi_{l}^{\prime }(x),\phi_{p}(x))v\psi_{k}(v),\psi_{q}(v))\\ & +\kappa \theta (\phi_{l}(x),\phi_{p}(x))(\psi_{k}^{\prime }(v),\psi_{q}(v))-\kappa (\phi_{l}(x),\phi_{p}(x))(v\psi_{k}^{\prime }(v),\psi_{q}(v))\\ & +\frac{1}{2}((\pi_{*}^{2}(t,x,v)x^{2}\phi_{l}^{\prime \prime }(x),\phi_{p}(x))v\psi_{k}(v),\psi_{q}(v))+\frac{1}{2}\zeta^{2}(\phi_{l}(x),\phi_{p}(x))(v\psi_{k}^{\prime \prime }(v),\psi_{j}(v))\\ & +\rho \zeta ((x\pi_{*}(t,x,v)\phi_{l}^{\prime }(x),\phi_{p}(x))v\psi_{k}^{\prime }(v),\psi_{q}(v))-\beta (\phi_{l}(x),\phi_{p}(x))(\psi_{k}(v),\psi_{q}(v))]\\ & -\lambda ((U(x-K)-\sum_{k=1}^{n_{2}}\sum_{l=1}^{n_{1}}V_{lk}^{i}\phi_{l}(x)\psi_{k}(v){)}^{+},\phi_{p}(x)),\psi_{q}(v))\}, \end{array}$$ (47)

where


$$\begin{array}{c} V_{0k}^{i}=0\;\text{and}\;V_{nk}^{i}=\Psi (t_{i},x_{\max}), \end{array}$$

and $\eta_{lk}=0$ or 1 will be specified later.

Applying GLL quadrature to (47) yields the following discrete system:


$$\begin{array}{cc} & V_{pq}^{i+1}-V_{pq}^{i}\\ = & \frac{\Delta t}{JJ^{\prime }\omega_{p}\omega_{q}}\{\sum_{l=0}^{n_{1}}\sum_{k=0}^{n_{2}}(\eta_{lk}V_{lk}^{i}+(1-\eta_{lk})V_{lk}^{i+1})[\frac{J^{\prime }}{J}(rx_{p}+Ax_{p}\pi_{pq}v_{q})A_{pl}M_{qk}\\ & +\kappa \theta (1-V_{q})M_{pl}A_{qk}+\frac{J^{\prime }}{2J}\pi_{pq}^{2}x_{p}^{2}v_{q}S_{pl}M_{qk}+\frac{J}{2J^{\prime }}\zeta^{2}v_{p}M_{pl}S_{qk}+\rho \zeta x_{p}\pi_{pq}v_{j}A_{pl}A_{qk}\\ & -\frac{\beta J^{\prime }}{J}M_{pl}M_{qk}]-\lambda JJ^{\prime }\omega_{p}\omega_{q}((U(x_{p}-K)-V_{pq}^{i})\}, \end{array}$$ (48)

where $\pi_{pq}=\pi_{*}(t_{i+1},x_{p},v_{q})$. Now let’s discuss the value of $\eta_{lk}$,


$$\begin{array}{cc} \eta_{lk}= & \left\{ \begin{array}{c} 0,\;{\left[{𝐐}_{i}^{\pi }\right]}_{lk}>0,\\ 1,\;{\left[{𝐐}_{i}^{\pi }\right]}_{lk}\leq 0. \end{array}\right. \end{array}$$

$$\begin{array}{cc} {\left[{𝐐}_{i}^{\pi }\right]}_{lk} & :=\frac{J^{\prime }}{J}(rx_{p}+Ax_{p}\pi_{pq}v_{q})A_{pl}M_{qk}+\kappa \theta (1-V_{q})M_{pl}A_{qk}\\ & +\frac{J^{\prime }}{2J}\pi_{pq}^{2}x_{p}^{2}v_{q}S_{pl}M_{qk}+\frac{J}{2J^{\prime }}\zeta^{2}v_{p}M_{pl}S_{qk}+\rho \zeta x_{I}\pi_{pq}v_{j}A_{pl}A_{qk}-\frac{\beta J^{\prime }}{J_{1}}M_{pl}M_{qk}. \end{array}$$

We apply the policy iteration scheme in Algorithm 1 to compute the solution of (48). The overall iteration logic is consistent, with only slight adjustments needed, and we thus skip detailed restatement.

### Numerical examples

In this section, we conduct numerical experiments to assess the performance of the proposed GLL spectral method for the penalized HJB equations. The results demonstrate linear convergence in time and exponential convergence in space. Furthermore, the exercise boundaries are accurately captured and visualized, and we verify the convergence of the penalized solution to that of (5) as the penalty parameter λ→∞.

**Example 0.1.** *This example employs two utility functions to examine the convergence of Algorithm 1: the power utility* $U(x-K)=\frac{x^{1/2}}{1/2}$ *and the non-HARA utility function* $U(x)=\frac{1}{3}H^{-3}(x)+H^{-1}(x)+xH(x)$*, where* $H(x)=\sqrt{2}(-1+\sqrt{1+4x}{)}^{-1/2}$ *(see* [29]*). To comprehensively evaluate the robustness of the numerical scheme, the values of the risk-free rate (r), return rate (*μ*), volatility of risky assets (*σ*), discount factor (*β*), and minimum wealth threshold value (K) in the HJB equation are specified into two distinct parameter sets:*

***Set I***: r=0.05, μ=0.1, σ=0.3, β=0.1, K=1.

***Set II***: r=0.03, μ=0.15, σ=0.2, β=0.08, K=1.

For the numerical experiment, we take $x_{\max}=10$, the initial wealth *x*_0_ = 1, and the investment horizon *T* = 0.1. Meanwhile, the iteration in Algorithm 1 stops when the tolerance reaches 10^–8^. The boundary conditions are specified below:


$$\begin{array}{c} V(t,x_{\max})=\phi (t,x_{\max})=e^{-\beta t}U(x_{\max}-K). \end{array}$$

Benchmark values are obtained via computations using a dense discretization with *n* = 64 spatial and *m* = 128000 temporal meshes, and a penalty parameter $\lambda =10^{3}$. Throughout the experiments, all relative errors are measured in the global *L*^2^-norm. Fig 1, which plots the data from Table 1, demonstrates that the GLL spectral method achieves exponential convergence in space under both parameter sets. Correspondingly, Fig 2 (based on Table 2) illustrates its linear convergence in time across different settings. The evolution of relative *L*^2^ errors with increasing λ, displayed in Fig 3, confirms the convergence of the penalized HJB solution to that of the original variational inequality (5) as λ→∞ for both Set I and Set II (Table 3).

**Table 1:** Spatial convergence of the GLL spectral method with respect to *n* (*m* = 2^14^, $\lambda =10^{3}$).

Utility	*n*	Error		Avg. Iterations		Time (sec)	
		Set I	Set II	Set I	Set II	Set I	Set II
**Power**	4	4.80E-01	4.81E-01	2.05	2.05	0.144	0.128
	8	3.43E-01	3.46E-01	3.00	3.00	0.257	0.258
	16	2.36E-01	2.41E-01	3.00	3.00	0.374	0.370
	32	1.57E-01	1.62E-01	3.00	3.00	0.819	0.826
	64	8.65E-02	1.00E-01	3.04	3.10	2.271	2.343
**Non-HARA**	4	5.04E-01	5.06E-01	2.05	3.00	0.145	0.229
	8	3.60E-01	3.64E-01	3.00	3.00	0.262	0.264
	16	2.48E-01	2.53E-01	3.00	3.00	0.376	0.371
	32	1.65E-01	1.70E-01	3.00	3.00	0.823	0.834
	64	9.10E-02	1.05E-01	3.06	3.13	2.269	2.373

**Table 2:** Temporal convergence of the GLL spectral method with respect to *m* (*n* = 64, $\lambda =10^{3}$).

Utility	*m*	Error		Avg. Iterations		Time (sec)	
		Set I	Set II	Set I	Set II	Set I	Set II
**Power**	2^14^	8.88E-07	2.24E-06	3.78	3.00	1.058	0.697
	2^15^	4.71E-07	1.04E-06	3.01	3.00	1.398	1.413
	2^16^	2.23E-07	4.47E-07	3.00	3.00	2.794	2.820
	2^17^	7.98E-08	1.49E-07	3.00	3.00	5.592	5.616
**Non-HARA**	2^14^	9.23E-07	3.75E-06	4.05	3.00	1.145	0.710
	2^15^	4.91E-07	1.75E-06	3.02	3.00	1.429	1.418
	2^16^	2.33E-07	7.50E-07	3.00	3.00	2.817	2.839
	2^17^	8.39E-08	2.50E-07	3.00	3.00	5.708	5.792

**Table 3:** Convergence of the penalized HJB equations to the original QVI with respect to λ (*n* = 64, *m* = 2^14^).

Utility	λ	Error		Avg. Iterations		Time (sec)	
		Set I	Set II	Set I	Set II	Set I	Set II
**Power**	4	2.28E-01	1.56E-01	3.43	3.50	2.237	2.237
	8	2.07E-01	1.49E-01	3.15	3.52	1.920	2.269
	16	1.64E-01	1.29E-01	3.45	3.47	2.185	2.224
	32	1.16E-01	8.26E-02	3.23	3.23	2.005	1.995
	64	6.82E-02	5.19E-02	3.11	3.01	1.892	1.801
	128	3.66E-02	2.70E-02	3.03	3.00	1.851	1.809
	256	1.77E-02	1.10E-02	3.01	3.00	1.808	1.856
	512	6.18E-03	4.73E-03	3.02	3.00	1.808	1.831
**Non-HARA**	4	2.46E-01	1.64E-01	3.40	3.54	2.270	2.253
	8	2.18E-01	1.56E-01	3.10	3.45	1.913	2.229
	16	1.72E-01	1.36E-01	3.25	3.34	2.037	2.126
	32	1.22E-01	8.68E-02	3.04	3.37	1.844	2.128
	64	7.17E-02	5.44E-02	3.03	3.01	1.832	1.815
	128	3.85E-02	2.83E-02	3.03	3.00	1.844	1.793
	256	1.86E-02	1.15E-02	3.01	3.00	1.836	1.814
	512	6.50E-03	5.07E-03	3.03	3.00	1.852	1.796

**Fig 1:**
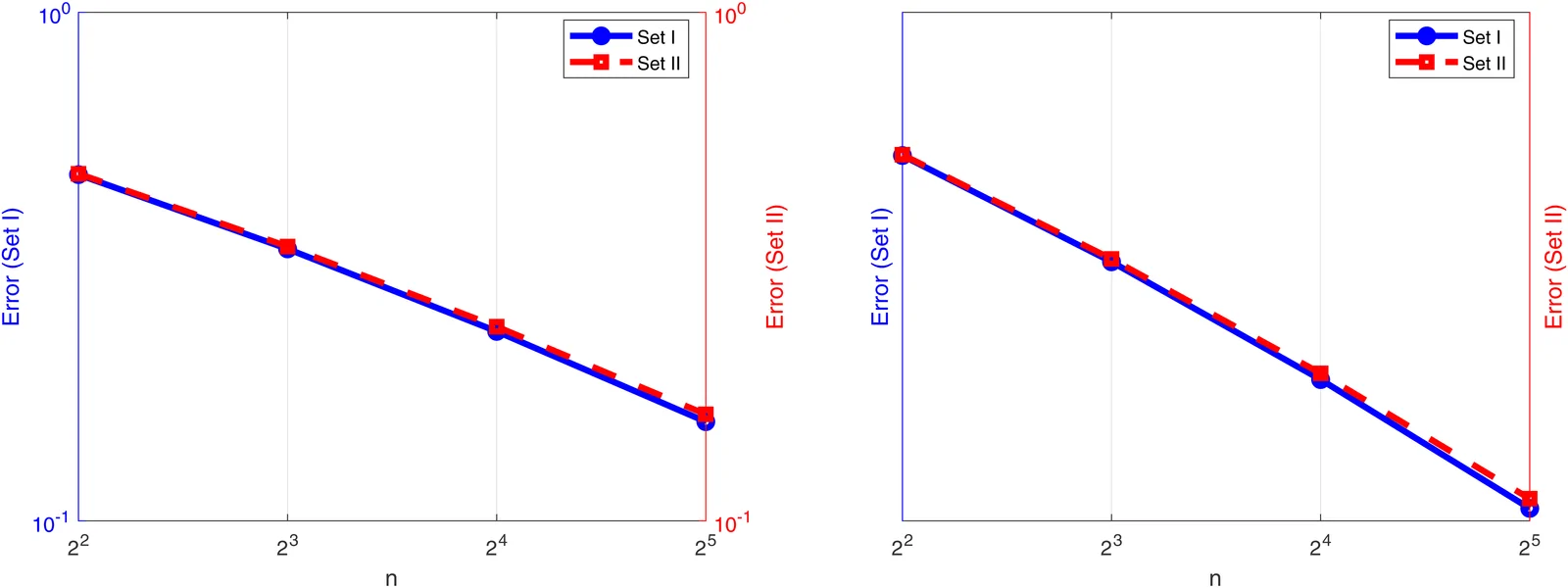
Convergence of penalized HJB equations toward the HJB variational inequality as *n* varies. (*m* = 2^14^), $\lambda =10^{3}$.

**Fig 2:**
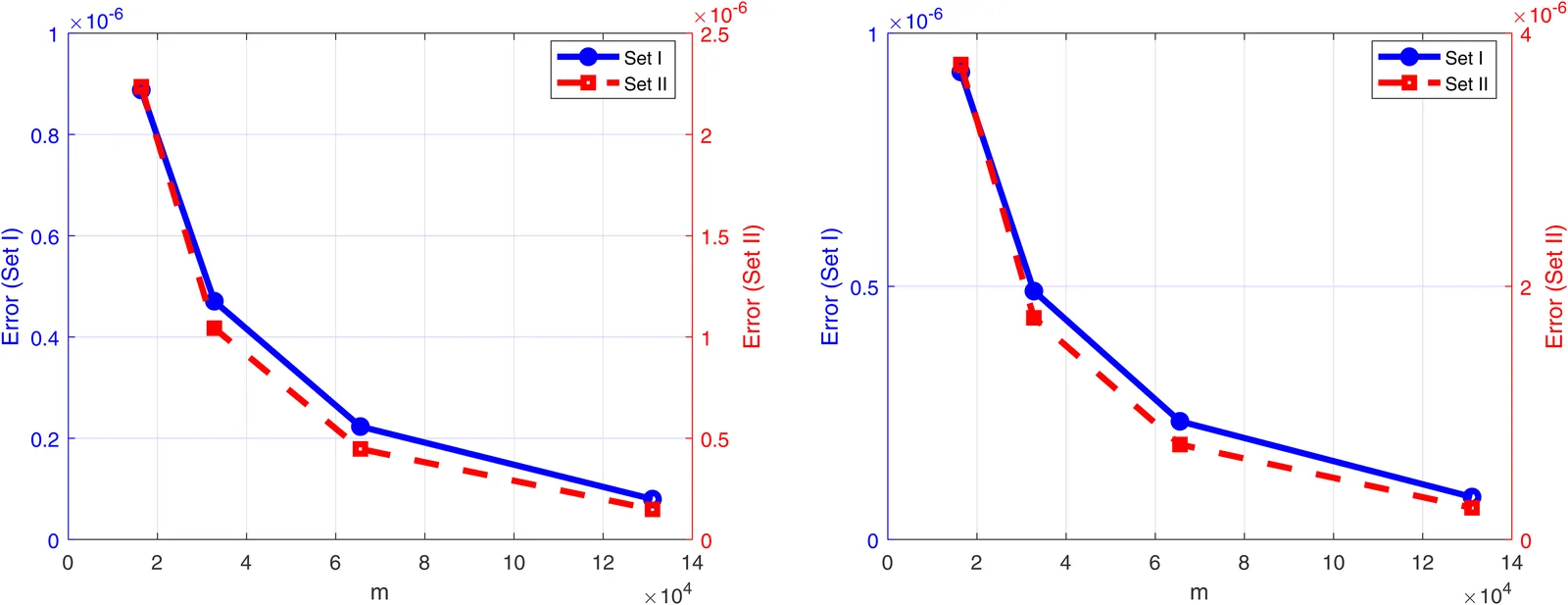
Convergence of penalized HJB equations toward the HJB variational inequality as *m* varies. (*n* = 64, $\lambda =10^{3}$).

**Fig 3:**
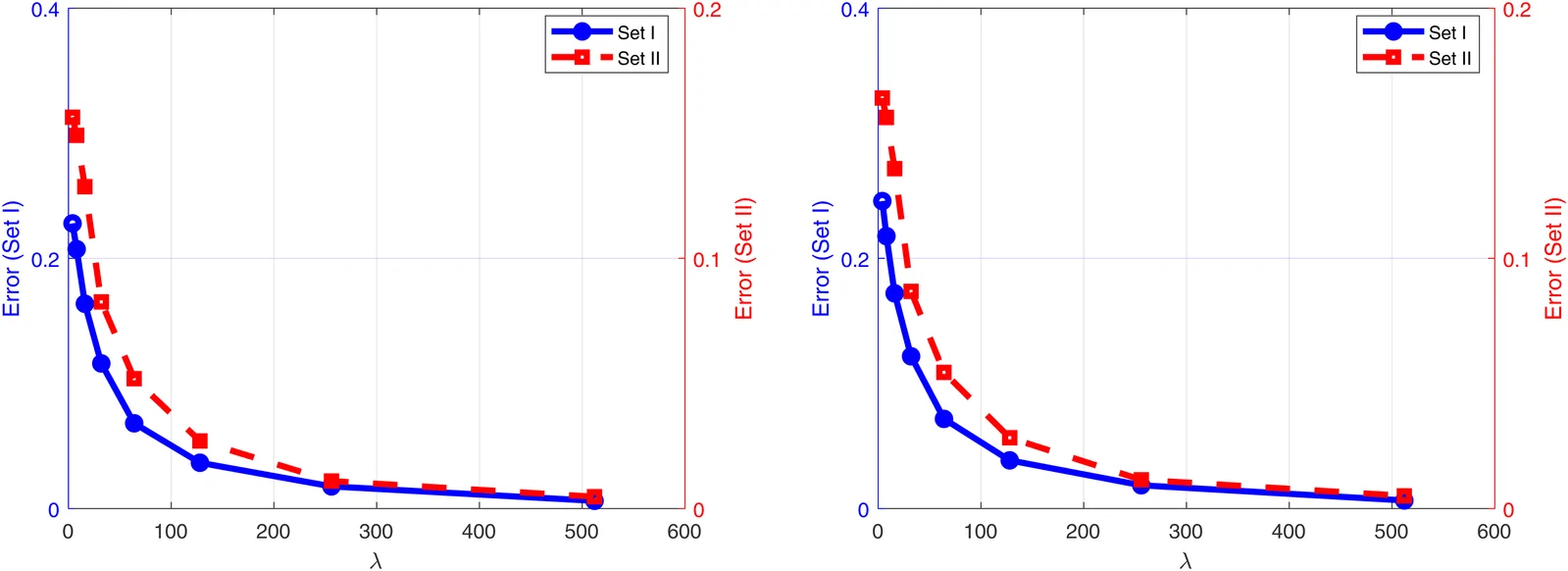
Convergence of penalized HJB equations toward the HJB variational inequality as λ varies. (*n* = 64, *m* = 2^14^).

**Example 0.2.** *This example extends the framework to a two-dimensional Heston stochastic volatility model to investigate the performance of the numerical method. The value function is governed by the wealth x and the variance v. We adopt the power utility function* $U(x-K)=\frac{x^{1/2}}{1/2}$ *with the minimum wealth threshold K = 1. The baseline parameters of the Heston model and the market are chosen as follows:*


$$\begin{array}{c} r=0.05,\;\mu =0.1,\;\beta =0.06,\;A=0.5,\;\kappa =0.3,\;\theta =0.02,\;\zeta =0.3,\;\rho =-0.7. \end{array}$$

For the numerical discretization and simulation, the spatial domain is chosen as $\left[x_{\min},x_{\max}]\times [v_{\min},v_{\max}]=[1,10]\times [10^{-7},0.6\right]$, the investment horizon is set to *T* = 0.1, and the inner-layer iteration tolerance of algorithm is 10^–8^. The reference benchmark solutions are computed using a refined mesh with *n*_1_ = 16, *n*_2_ = 16, *m* = 128, and a penalty parameter λ=900.

To comprehensively analyze the convergence behavior of the proposed GLL spectral scheme, all relative errors are evaluated in the global *L*^2^-norm. Similar to the one-dimensional case, Table 4 and Fig 4 demonstrates the method’s high-order spatial convergence, linear temporal convergence, and asymptotic convergence as the penalty parameter λ increases.

**Table 4:** Sensitivity analysis of the GLL spectral method for the 2D Heston QVI with respect to $n_{1},n_{2},m$, and λ.

Parameter: $n_{1}$				Parameter: $n_{2}$			
*n*_1_	Error	Avg. Iter	Time (sec)	*n*_2_	Error	Avg. Iter	Time (sec)
2	1.00E-01	1.00	0.028	2	1.07E-04	1.02	0.076
4	4.11E-02	1.79	0.046	4	9.81E-05	1.00	0.146
6	2.31E-02	1.14	0.043	6	8.00E-05	1.00	0.225
8	1.47E-02	1.02	0.056	8	6.56E-05	1.00	0.343
10	9.62E-03	1.01	0.092	10	5.10E-05	1.00	0.489
12	6.04E-03	1.01	0.109	12	3.58E-05	1.00	0.818
14	3.09E-03	1.01	0.138	14	1.88E-05	1.00	1.036
**Parameter: m**				**Parameter: λ**			
*m*	**Error**	**Avg. Iter**	**Time (sec)**	λ	**Error**	**Avg. Iter**	**Time (sec)**
2^8^	6.43E-05	1.01	0.173	4	1.48E-03	1.29	0.182
2^9^	3.74E-05	1.00	0.289	8	1.13E-03	1.21	0.166
2^10^	1.87E-05	1.00	0.564	16	7.75E-04	1.15	0.157
2^11^	9.26E-06	1.00	1.120	32	4.52E-04	1.10	0.149
2^12^	4.49E-06	1.00	2.238	64	2.32E-04	1.06	0.144
2^13^	2.10E-06	1.00	4.432	128	1.13E-04	1.04	0.140
2^14^	9.00E-07	1.00	8.890	256	4.86E-05	1.02	0.141
2^15^	3.00E-07	1.00	17.784	512	1.63E-05	1.01	0.139

**Fig 4:**
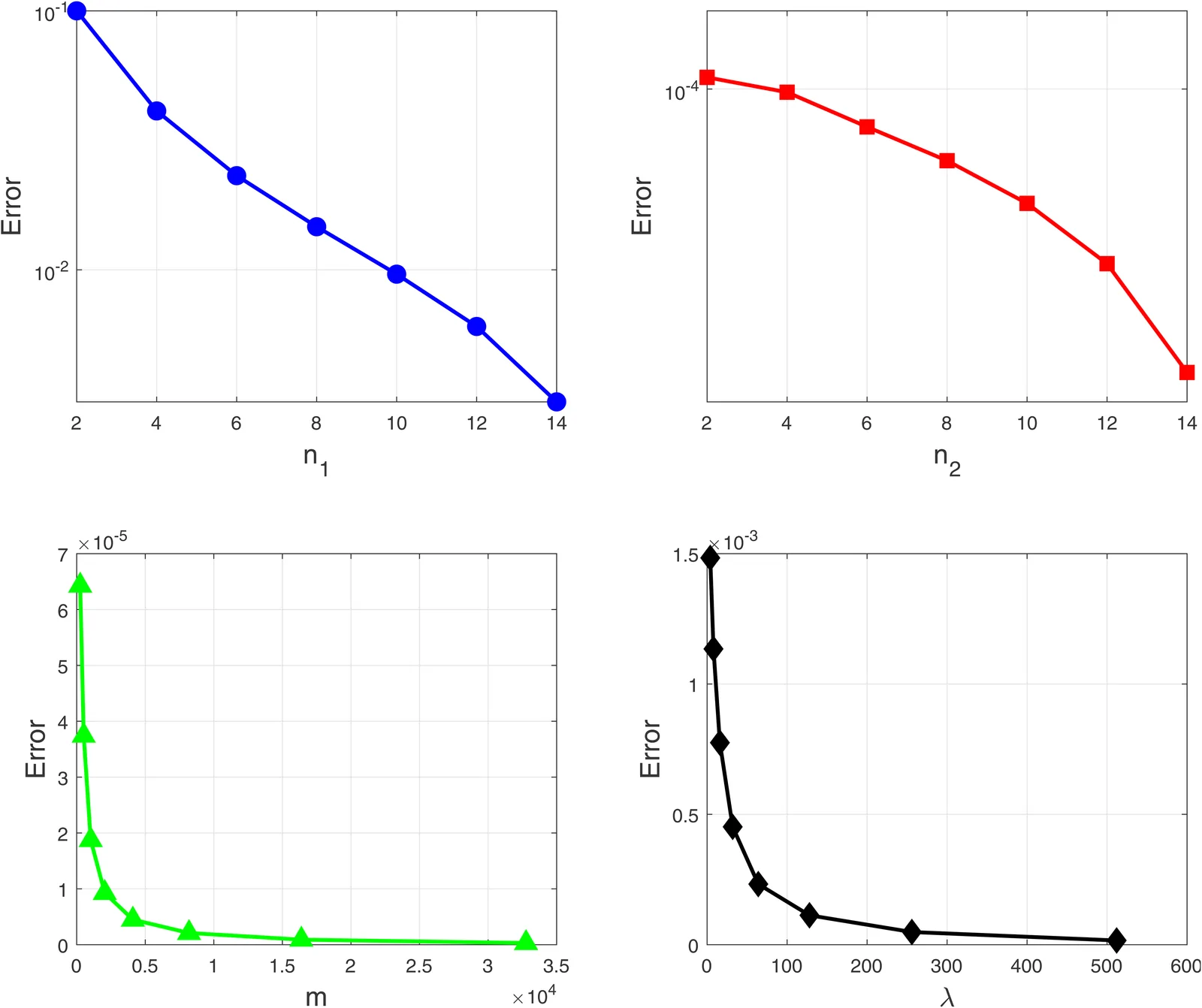
The relative *L*^2^-norm errors with respect to the spatial nodes ($n_{1},n_{2}$), temporal steps (*m*), and the penalty parameter (λ).

## Conclusions

In summary, this work addresses a utility maximization problem involving optimal stopping, mathematically formulated as a HJB QVI. We develop a novel computational framework that integrates GLL spectral discretization with a penalization method, implemented via a policy iteration scheme. The convergence of the penalized system is theoretically established. Finally, the efficacy and precision of the proposed methodology are verified through a comprehensive set of numerical experiments.

## Supporting information

S1 File Source code used to reproduce the numerical experiments and results reported in this study. (RAR)
